# Case of Epilepsy Cured by Iodine

**Published:** 1831

**Authors:** 


### XXIV.

### CASE OF EPILEPSY CURED BY IODINE.

DR. FRANKLIN, of New York, has published the following case, which we shall lay before our readers.

Feb. 28th, 1831. O. B. the subject of this complaint, early displayed great precocity of intellect, and was endowed with a frame proportionate in vigour to his intellectual capacity; in complexion fair; temperament sanguine; quickly impelled to anger, but easily soothed; indignant at insult, but alive to kindness; disposed to friendship, and discriminating in his selections; ambitious beyond his years; with a memory almost intuitive. No obstacle seemed equal to prevent his advance to the *ultima thule* of any profession to which inclination should direct him. The expectations of his friends were damped by an attack of epilepsy, the incipient symptoms and progress of which I will now proceed to relate.

His nervous system from early boyhood indicated an excessive irritability to external impressions; whether this was congenital, or induced, as his mother surmised, by excessive doses of calomel prescribed by a medical gentleman in the South, during a fit of sickness,* I am unable to decide.

When about eight years of age the attention of his parents was directed to the position he would assume when injured by any external agent. If his toes struck against a stone, or his fingers were irritated by any sharp instrument, as a pin or a needle, his eyes protruded, the muscles of his mouth were agitated, his arms contracted and elevated towards his head, and he would stand as though transfixed with horror at the sight of some terrible object. As these paroxysms subsided in a few seconds, they were rather attributed to a foolish habit he had acquired than to any disease, and under this impression he was frequently reproved by his parents, but without avail.

About this time he escaped their cognizance by being sent to school in the country, where from subsequent information it appears the paroxysms increased rapidly in severity and number. He would then occasionally for the first time, when unexpectedly struck by his playmates, or upon the receipt of any injury, fall to the ground without the ability of resisting the impulse. His teacher having directed the boys to dash water in his face to bring him to, they would frequently induce a fit by striking or pricking him for the sport afforded in throwing water. His countenance, hitherto indicative of vivacity and intelligence, now began to assume the vacancy so peculiar to this complaint, and his mental faculties, hitherto vigorous, began to be impaired.

After an absence of a year he returned home, when his altered appearance, and the frequency of his fits, immediately attracted attention and excited great alarm. The most trivial injury induced a fit, and he would now invariably fall, bruising and lacerating himself dreadfully, always falling upon his face or the back of his head, generally the latter, and that so frequently as to establish a natural issue.

It was now for the first time discovered that these fits occurred during sleep; at these times he would foam at the mouth and roll his eyes; his hands and arms would be spasmodically contracted, and drawn towards his head. He would gasp for breath, and in his worst fits would turn black in the face. These paroxysms subsided in four or five minutes, leaving him very languid and exhausted.

He said he was conscious of what was passing about him, but could not articulate. The pain he described as that caused by two bones meeting and rubbing in his chest. The sense of his situation operated so acutely upon him that he preferred death to life. His appetite was good; the food was regularly assimilated; he complained of no pain in any part of the system during the intervals of paroxysm.

Such was the state of the patient when I commenced the treatment. I ordered the *Ecioprotic mixture* in quan-

* Bilious fever, at Florence.

ties sufficient to keep the bowels soluble, and the *tinct. of iodine*, 40 grs. to the oz. gtt. v. to be taken three times a day. This was gradually increased with but little success until he took 300 per diem, or 100 three times a day. At this time symptoms of amendment displayed themselves in the mildness of the night fits, and their diminished number. Flattered by this improvement, I continued the above-mentioned quantity for about one month, the system daily becoming less and less obnoxious to the disease, until I am happy to say, every vestige of the complaint disappeared. From that time to the present, a period of three months, he has enjoyed uninterrupted health. In conclusion permit me to add, instead of emaciation as I had a right to expect, by reduction of diet and the medicines taken, he actually gained flesh.—*Lancet.*

*Remarks.* We doubt very much the propriety of calling the above disease a case of epilepsy. The anomalous features of the attacks, and the circumstance of *consciousness* remaining during the paroxysms, have raised our doubts. Be this as it may, the enormous quantity of iodine taken, is enough to astonish us, if not to create an apprehension that the medicine was totally inert. The want of all apparent effect from the prodigious doses of iodine, excepting the fattening process which took place during its exhibition, is another stumbling-block which we cannot easily get over.

